# Association between intimate partner violence and mentally unhealthy days in women in the U.S

**DOI:** 10.21633/jgpha.6.2s12

**Published:** 2016

**Authors:** Danielle Broadnax, Reinetta Thompson Waldrop, Mechelle D. Claridy, Elaine Archie Booker, Ernest Alema-Mensah

**Affiliations:** 1 Master of Public Health Program, Morehouse School of Medicine, Atlanta, GA; 2 Department of Community Health and Preventative Medicine, Morehouse School of Medicine, Atlanta, GA

**Keywords:** intimate partner violence, mental health, women’s health

## Abstract

**Background:**

In the United States (U.S.), intimate partner violence (IPV) is a serious public health concern, mainly affecting the health and well-being of women. The objective of this study was to identify the IPV and socio-demographic factors associated with mentally unhealthy days among women in the U.S. of ages ≥18 years.

**Methods:**

Data for this study were obtained from the 2007 Behavioral Risk Factor Surveillance System. Multivariable analyses were used to estimate adjusted odds ratios (AORs) and 95% confidence intervals (95% CIs) for factors associated with IPV and 14 or more mentally unhealthy days per month. Analyses were conducted using SAS 9.3.

**Results:**

The analyses show that the following factors increase the likelihood of self-reported 14 or more mentally unhealthy days: having a high school level of education or less (AOR: 1.732; 95% CI: 1.415–2.119) and having an income < $50,000. In addition, experiencing IPV such as: ever being threatened by a sex partner (AOR: 1.499; 95% CI: 1.264–1.779); having a sex partner ever attempt violence (AOR: 1.461; 95% CI: 1.224–1.743); having a sex partner ever become violent (AOR: 1.541; 95% CI: 1.303–1.823); and ever having unwanted sex with a partner (AOR: 1.929; 95% CI: 1.584–2.350) also increased the likelihood of self-reported 14 or more mentally unhealthy days per month.

**Conclusions:**

The results indicate that, for women in the U.S., IPV and socio-demographic factors have an effect on self-reported 14 or more mentally unhealthy days. Improving access to services that offer protection and guidance for women abused by their intimate partner could decrease the likelihood of selfreported 14 or more mentally unhealthy days and long-term negative mental health outcomes among women.

## INTRODUCTION

Intimate partner violence (IPV) and mental health issues are pressing public health concerns. During every minute, an average of 24 people are raped, physically harmed, or stalked by their partner (Spivak et al., 2014). These numbers do not completely reflect the magnitude of this problem, because many occurrences of IPV are not reported by victims (Spivak et al., 2014). In 2010, 1,295 deaths resulted from IPV, and, in that year, IPV accounted for 10% of all homicidal fatalities. Women are almost three times more likely to incur injury from IPV than men, and they are also twice as likely to die from IPV (Spivak et al., 2014).

Women who are victims of IPV have a higher prevalence of adverse physical and mental health outcomes than women who are not involved in IPV (Black et al., 2011). Among those women who consider their overall mental or physical health to be poor, the percentage is almost three times higher for women with a history of IPV than women who have not experienced IPV (Black et al., 2011).

The long-term outcomes of IPV usually consist of those affecting the mental health of women. Various studies have supported the notion that women who are victims of IPV experience mental health issues due to this exposure (Cerulli, Poleshuck, Raimondi, Veale, & Chin, 2012; Karakurt, Smith, & Whiting, 2014; Lipsky, Kernic, Qiu, Wright, & Hasin, 2014; Messing, Thaller, & Bagwell, 2014; Pico-Alfonso et al., 2006). Lipsky et al. (2014) agree that post-traumatic stress disorder (PTSD) and depression are associated with IPV victimization. Among women, being a victim of physical IPV is associated with an increased risk for poor health, symptoms of depression, and development of a chronic mental illness (Lipsky et al., 2014). In a study by Cerulli et al. (2012), women described that they experienced depression, anxiety, panic attacks, and even flashbacks of IPV incidents. These psychological consequences led some victims to contemplate suicide (Cerulli et al., 2012). This claim is supported by Messing et al. (2014), who state that women who experienced both physical and sexual violence from a partner are more likely to experience depression, PTSD, and suicidal ideation. Pico-Alfonso et al. (2006) suggests that the symptoms of depression and PTSD are comorbid.

There is a substantial amount of literature regarding IPV and related adverse mental health outcomes, but there is a need for more information examining the association in a representative sample of women in the United States (U.S). The current study is the first to use the 2007 BRFSS IPV module to dichotomize mentally unhealthy days (1–13 mentally unhealthy days and 14 or more mentally unhealthy days per month) to examine its association with IPV by controlling for variables such as race, education, age, and income level. The results from this study will add to the current body of literature and bring awareness to the mental health issues that women face when they become victims of IPV. The objective was to examine the association between IPV and 14 or more mentally unhealthy days per month among women in the U.S.

## METHODS

### Study Design and Data Source

To assess the association between IPV and 14 or more mentally unhealthy days among women aged 18 years and over in the U.S., data from the 2007 Behavioral Risk Factor Surveillance System (BRFSS) survey (N=91,287) were analyzed. This was the latest year that the survey used the optional Intimate Partner Violence Module. BRFSS is a state-based, cross-sectional survey designed to measure behavioral risk factors within the adult population of those aged 18 years and older residing in households (CDC, 2013). This survey is the largest regularly administered health survey in the world due to its completion of more than 400,000 interviews yearly. Data are collected via telephone in 50 states, the District of Columbia, Guam, the Virgin Islands, and Puerto Rico (CDC, 2013). Participants are asked a series of questions from the core component of the questionnaire, which requires an average of ten minutes, and from the optional modules and state-added questions, which take an additional five to ten minutes (CDC, 2013).

### Participants

BRFSS selects individuals randomly by dialing household telephone numbers and interviews only one participant per household (CDC, 2013). The present study used data from adult female respondents age 18 and over who lived in the U.S. The inclusion criteria were that the participants must have responded to both the 2007 BRFSS survey and the optional Intimate Partner Violence Module for that survey year.

### Measures

All measures were based on self-reported data obtained from the 2007 BRFSS. To evaluate the exposure/independent variable, IPV, variables from the Intimate Partner Violence Module were used. The following variables were used to assess IPV exposure:

1. “Has an intimate partner ever threatened you with physical violence? This includes threatening to hit, slap, push, kick, or hurt you in any way.”
2. “Has an intimate partner ever attempted physical violence against you? This includes times when they tried to hit, slap, push, kick, or otherwise hurt you, but they were not able to.”
3. “Has an intimate partner ever hit, slapped, pushed, kicked, or hurt you in any way?”
4. “Have you ever experienced any unwanted sex by a current or former intimate partner?”
5. “In the past 12 months, have you experienced any physical violence or had unwanted sex with an intimate partner?”

The question relating to mentally unhealthy days is one of four core health-related quality of life measures that have been monitored by states using the BRFSS. Responses to the following question were used to determine the prevalence of mentally unhealthy days among women: “Now thinking about your mental health, which includes stress, depression, and problems with emotions, for how many days during the past 30 days was your mental health not good?” The response options were “_ _ number of days,” “none,” “don’t know/not sure,” or “refused.” Only those records with “the number of days” were included in the analysis. The items measured in days were dichotomized at a cut-off value of ≥ 14 days of poor health in the past month versus ≤ 13 (CDC, 2016). This 14-day minimum period was selected because supporting documentation from the CDC define a similar period that is often used by clinicians and clinical researchers as a marker for clinical depression and anxiety disorders (CDC, 2016). If a woman, within a month, experiences 14 or more days in which her mental health is not good, she is classified as having frequent mental distress (CDC, 2000).

Additional covariates considered were race, education, age, and income level. The Institutional Review Board at Morehouse School of Medicine approved this study.

### Procedures/Analysis

Univariate analyses were used to describe the frequency and proportion of selected characteristics (race, education, age, and income level). Bivariate analyses were conducted with chi-square tests to assess the association between mentally unhealthy days, socio-demographic factors, and IPV. Multivariable logistic regressions were used to estimate adjusted odds ratios (ORs) and 95% confidence interval (CIs) for factors associated with mentally unhealthy days. To maximize statistical power in the analyses, predictors found to be non-significant (p value > 0.05) were removed. The predictors of mentally unhealthy days with a p value of ≤ 0.05 in the bivariate analyses were entered into the model. For mentally unhealthy days, these variables were race, age, education, income, and IPV. Since the IPV variable “In the past 12 months, have you experienced any physical violence or had unwanted sex with an intimate partner” was not significant in bivariate analyses, it was not included in multivariable analyses. Bivariate and multivariable analyses excluded persons with responses that were missing or recorded as “don’t know/not sure” or “refused.” A 2-sided p value of ≤ 0.05 was considered to indicate statistical significance. Analyses were conducted using SAS version 9.3.

## RESULTS

A summary of the number of mentally unhealthy days by the sociodemographic characteristics and IPV for women (N=91,287) aged ≥18 years and interviewed in the 2007 BRFSS are presented in Tables 1 and 2. Of the participants who responded to the question: “Now thinking about your mental health, which includes stress, depression, and problems with emotions, for how many days during the past 30 days was your mental health not good?” 30,257 (33.14%) reported having 14 or more mentally unhealthy days, and 61,030 (66.86%) reported having 1–13 mentally unhealthy days. Of the respondents who had 14 or more mentally unhealthy days, 80.97% were White; and 47.02% were between 45–64 years of age. Most respondents (50.29%) had a high school education or less; and (49.27%) had an income < $24,999 (Table 1). In addition, 32.54% reported that their *sex partner had ever threatened violence*; 28.70% reported that their *sex partner had ever attempted violence*; 34.91% reported that their *sex partner had been violent*; 23.22% reported that they *had unwanted sex with their partner*; and 8.18% reported that they *had unwanted sex or experienced physical violence with their partner within the last 12 months* (Table 2).

Table 3 displays the results of multivariable logistic regressions for 14 or more mentally unhealthy days. In these analyses, a factor associated with lower odds of experiencing 14 or more mentally unhealthy days included being between the ages of 65 and 74 (AOR: 0.743; 95% CI: 0.554–0.995). Factors associated with higher odds of 14 or more mentally unhealthy days included having a high school level of education or less (AOR: 1.732; 95% CI: 1.415–2.119), having an income < $24,999 (AOR: 2.883; 95% CI: 2.263–3.674), and having an income from $25,000 to $49,999 (AOR: 1.517; 95% CI: 1.202–1.914). In addition, experiencing IPV such as: *ever being threatened by a sex partner* (AOR: 1.499; 95% CI: 1.264–1.779); *having a sex partner ever attempt violence* (AOR: 1.461; 95% CI: 1.224–1.743); *having a sex partner ever become violent* (AOR: 1.541; 95% CI: 1.303–1.823); and *ever having unwanted sex with a partner* (AOR: 1.929; 95% CI: 1.584–2.350) also increased the likelihood of self-reported 14 or more mentally unhealthy days.

## DISCUSSION

The objective of this investigation was to examine the association between 14 or more mentally unhealthy days and IPV among women in the U.S. All sociodemographic and IPV variables, except race and unwanted sex or physical violence with a partner within the last 12 months, were significantly associated with 14 or more mentally unhealthy days. Relative to women between the ages of 18 and 44, those women who were between 65 and 74 years of age were less likely to self-report 14 or more mentally unhealthy days. In support of this finding, according to the Morbidity and Mortality Weekly Report (MMWR) prepared by the CDC, a report by Zahran et al. (2005) showed that, for the 1993–2001 BRFSS surveys, younger adults reported the highest number of mentally unhealthy days. As age increased, the number of mentally unhealthy days decreased (Zahran et al., 2005).

Concerning income, those women who had an annual household income of $49,999 or less were more likely to self-report 14 or more mentally unhealthy days than women who had an annual household income of $75,000 or more. A previous study also showed this association (Zahran et al., 2005). Additionally, women who had a high school level of education or less were more likely to report 14 or more mentally unhealthy days than women who were college graduates. Similarly, the results reported by Zahran et al. (2005) showed that there were higher percentages of those with less than a high school education reporting 14 or more mentally unhealthy days. Nevertheless, those who had a high school level of education reported more mentally unhealthy days than those who had higher education (Zahran et al., 2005).

Results from the present study show that women who had a sex partner *ever threaten violence against them*, a sex partner *ever attempt violence against them*, a sex partner *ever become violent* or *had unwanted sex with a partner* self-reported 14 or more mentally unhealthy days more than women who had not experienced IPV in these forms. Being threatened by a sexual partner is a form of psychological abuse experienced by IPV victims (Rogers & Follingstad, 2014). Rogers and Follingstad (2014) assessed whether or not psychological abuse predicted mental health outcomes and concluded that women can report symptoms of anxiety, depression, and even suicidal ideation if they are threatened by their partner. In regard to physical violence, having a sexual partner ever attempt to become violent or become violent has a direct effect on the mental health of female victims (Karakurt et al., 2014). The findings regarding physical violence are supported by results of a study by Karakurt et al. (2014) showing that women who reported being physically abused by an intimate partner also reported mental health issues, including depression and other symptoms related to mental health. Concerning sexual violence, the results from a study by Coker et al. (2002) showed that women who have experienced sexual violence by an intimate partner are at greater risk for experiencing adverse mental health outcomes. For female victims, all three forms, psychological, physical, and sexual IPV, were associated with adverse self-perceived mental health consequences (Coker et al., 2002).

There are several strengths of the current study. It provides information on the effect of IPV on 14 or more mentally unhealthy days in women in the U.S. It also offers other explanations and evidence for the association between sociodemographic and IPV variables and mental health. Further, the present study uses data from the BRFSS, allowing the results to be generalizable to the U.S. population of female victims of IPV. A limitation is the use of selfreported data. Additionally, four of the five IPV factors did not include a timeframe associated with experiencing IPV. Thus, recall bias could be a limitation.

## CONCLUSIONS

Overall, 33% of the women in the study sample reported experiencing 14 or more mentally unhealthy days in the past 30 days. Mental health encompasses various aspects that may lead a woman who has been victimized by her intimate partner to consider her mental health poor. These indicators of poor mental health include depression, PTSD, suicidal ideation, sleep deprivation, and self-esteem issues (Cerulli et al., 2012; Karakurt et al., 2014; Lipsky et al., 2014; Messing et al., 2014; Pico-Alfonso et al., 2006). Women who are affected by this form of violence should seek help through counseling to obtain options for removing themselves from victimizing situations. The results of this study show the need for policies centered on the development of interventions that focus on mental health for those who have experienced IPV. Also, intervention programs can be created to provide information that will help all women, despite their race/ethnicity, socioeconomic status, or age to avoid becoming victims of IPV.

## Figures and Tables

**Table 1:** Number and percentage* of mentally unhealthy days by select characteristics: 2007 BRFSS, United States

Characteristics	Mentally unhealthy days (N=91,287)				p-value
All women	1–13 days		14 or more		
	n	%	n	%	
	61030	66.86	30257	33.14	
**Race**					p<0.0001
**White**	51113	83.75	24496	80.97	
**Black or African American**	5544	9.08	3198	10.57	
**Asian**	935	1.53	279	0.92	
**Native Hawaiian or Other Pacific Islander**	291	0.48	149	0.49	
**American Indian or Alaska Native**	1078	1.77	794	2.62	
**Other**	2066	3.39	1337	4.42	
**Total**	61027	100	30253	100	
**Age group**					p<0.0001
**18–44**	24499	42.13	10376	36.03	
**45–64**	24532	42.19	13543	47.02	
**65–74**	5302	9.12	2811	9.76	
**75 and above**	3817	6.56	2071	7.19	
**Total**	58150	100	28801	100	
**Level of education**					p<0.0001
**High School or less**	21240	34.86	15185	50.29	
**Some College**	17527	28.36	8866	29.36	
**College Graduate**	22166	36.38	6146	20.35	
**Total**	60933	100	30197	100	
**Level of income**					p<0.0001
**Less than $24,999**	14804	27.20	12931	49.27	
**$25,000 to $49,999**	15867	29.15	6828	26.02	
**$50,000 to $74,999**	9904	18.20	3105	11.83	
**$75,000 or more**	13850	25.45	3382	12.89	
**Total**	54425	100	26246	100	

* Frequencies may be different due to missing values.

**Table 2:** Number and percentage* of mentally unhealthy days by intimate partner violence: 2007 BRFSS, United States

Characteristics	Mentally unhealthy days (N=91,287)				p-value
All women	1–13 days		14 or more		
	n	%	n	%	
	61030	66.86	30257	33.14	
**Sex Partner Ever Threatened Violence**					p<0.0001
**Yes**	556	21.02	398	32.54	
**No**	2089	78.98	825	67.46	
**Total**	2645	100	1223	100	
**Sex Partner Ever Attempted Violence**					p<0.0001
**Yes**	501	18.94	351	28.70	
**No**	2144	81.06	872	71.30	
**Total**	2645	100	1223	100	
**Sex Partner Ever Violent**					p<0.0001
**Yes**	587	22.20	427	34.91	
**No**	2057	77.80	796	65.09	
**Total**	2644	100	1223	100	
**Ever Had Unwanted Sex With Partner**					p<0.0001
**Yes**	331	12.52	284	23.22	
**No**	2312	87.48	939	76.78	
**Total**	2643	100	1223	100	
**Unwanted Sex or Phys Violence With Partner Last 12 Mos.**					p=0.0505
**Yes**	37	5.33	40	8.18	
**No**	657	94.67	449	91.82	
**Total**	694	100	489	100	

* Frequencies may be different due to missing values

**Table 3:** Multivariable Analysis of factors associated with 14 or more mentally unhealthy days and intimate partner violence: 2007 BRFSS, United States

Characteristics	Mentally unhealthy days (14 or more)
	Adjusted OR
	(95% CI)
**Race**	
**White**	Ref
**Black or African American**	0.883 (0.601–1.297)
**Asian**	0.954 (0.734–1.240)
**Native Hawaiian or Other Pacific Islander**	0.964 (0.692–1.344)
**American Indian or Alaska Native**	1.116 (0.509–2.446)
**Other**	0.808 (0.514–1.269)
**Age group**	
**18–44**	Ref
**45**–**64**	1.156 (0.982–1.360)
**65**–**74**	0.743 (0.554–0.995)
**75 and above**	0.750 (0.514–1.094)
**Level of education**	
**College Graduate**	Ref
**Some College**	1.150 (0.933–1.417)
**High School or Less**	1.732 (1.415–2.119)
**Annual Household Income**	
**$75,000 or more**	Ref
**$50,000 to $74,999**	1.249 (0.957–1.630)
**$25,000 to $49,999**	1.517 (1.202–1.914)
**Less than $24,999**	2.883 (2.263–3.674)
**Sex Partner Ever Threatened Violence**	
**No**	Ref
**Yes**	1.499 (1.264–1.779)
**Sex Partner Ever Attempted Violence**	
**No**	Ref
**Yes**	1.461 (1.224–1.743)
**Sex Partner Ever Violent**	
**No**	Ref
**Yes**	1.541 (1.303–1.823)
**Ever Had Unwanted Sex W/ Partner**	
**No**	Ref
**Yes**	1.929 (1.584–2.350)

## Statement of Student-Mentor Relationship

The lead author for this report, Danielle Broadnax, completed this work as her Master of Public Health Thesis at Morehouse School of Medicine. The co-authors, Reinetta Thompson Waldrop, Mechelle D. Claridy, Elaine Archie Booker, and Ernest Alema-Mensah, serve as her mentors.

## References

[R1] Black MC, Basile KC, Breiding MJ, Smith SG, Walters ML, Merrick MT, Stevens MR (2011). The National Intimate Partner and Sexual Violence Survey (NISVS): 2010 Summary Report.

[R2] Centers for Disease Control and Prevention (CDC) (2000). Measuring healthy days.

[R3] Centers for Disease Control and Prevention (CDC) (2013). BRFSS 2007 survey data and documentation.

[R4] Centers for Disease Control and Prevention (CDC) (2016). Health-related quality of life (HRQOL), frequently asked questions.

[R5] Cerulli C, Poleshuck E, Raimondi C, Veale S, Chin N (2012). “What fresh hell is this?” Victims of intimate partner violence describe their experiences of abuse, pain, and depression. Journal of Family Violence.

[R6] Coker AL, Smith PH, Thompson MP, McKeown RE, Bethea L, Davis KE (2002). Social support protects against the negative effects of partner violence on mental health. Journal of Women’s Health & Gender-Based Medicine.

[R7] Karakurt G, Smith D, Whiting J (2014). Impact of intimate partner violence on women’s mental health. Journal of Family Violence.

[R8] Lipsky S, Kernic MA, Qiu Q, Wright C, Hasin DS (2014). A two-way street for alcohol use and partner violence: Who’s driving it?. Journal of Family Violence.

[R9] Messing JT, Thaller J, Bagwell M (2014). Factors related to sexual abuse and forced sex in a sample of women experiencing IPV. Health and Social Work.

[R10] Pico-Alfonso MA, Garcia-Linares MI, Celda-Navarro N, Blasco-Ros C, Echeburua E, Martinez M (2006). The impact of physical, psychological, and sexual intimate male partner violence on women’s mental health: Depressive symptoms, posttraumatic stress disorder, state anxiety, and suicide. Journal of Women’s Health.

[R11] Rogers MJ, Follingstad DR (2014). Women’s exposure to psychological abuse: Does that experience predict mental health outcomes?. Journal of Family Violence.

[R12] Spivak HR, Jenkins EL, VanAudenhove K, Lee D, Kelly M, Iskander J (2014). CDC Grand Rounds: A public health approach to prevention of intimate partner violence. Morbidity and Mortality Weekly Report.

[R13] Zahran HS, Kobau R, Moriarty DG, Zack MM, Holt J, Donehoo R (2005). Health related quality of life surveillance—United States, 1993— 2002. Morbidity and Mortality Weekly Report.

